# Risk factors, healthcare-seeking and sexual behaviour among patients with genital ulcers in Zambia

**DOI:** 10.1186/1471-2458-12-407

**Published:** 2012-06-06

**Authors:** Mpundu Makasa, Knut Fylkesnes, Ingvild F Sandøy

**Affiliations:** 1 Lusaka District Health Management Team, Ministry of Health, Lusaka, Zambia; 2 Centre for International Health, University of Bergen, P. O. Box 7804, Bergen, NO-5020, Norway

**Keywords:** Genital ulcers (GUs), Inconsistent condom use, Care-seeking, Predictors, Zambia

## Abstract

**Background:**

Genital ulcers (GU) are associated with an increased risk of HIV transmission. Understanding risk factors for genital ulcers and sexual behaviour patterns after onset of symptoms and health seeking behaviour among GU-patients can provide useful information to aid design effective prevention strategies for genital ulcers. We investigated risk factors of self-reported GUs and care-seeking in the general population, and assessed GU patients regarding past care-seeking, recent sexual behaviour and partner awareness of the disease.

**Methods:**

We analysed national data on genital ulcers from the 2007 Zambia Demographic and Health Survey, and data from a cross-sectional survey of genital ulcer patients from primary health care facilities in Lusaka, Zambia.

**Results:**

The prevalence of GU in 2007 in the general population of Lusaka was 3.6%. Important predictors for genital ulcers were age 25–29 years, being widowed/separated/divorced and having a high number of life-time sexual partners. No differences in care-seeking were observed by residence, wealth and gender, and 60% of the respondents sought care from public health facilities. Among patients with GUs in Lusaka, 14% sought care >2 weeks after symptom onset. Forty-two percent were not aware of their HIV status, 57% reported sex after onset of symptoms and only 15% reported consistent condom use.

**Conclusions:**

Low awareness of HIV status despite high probability of being infected and low condom use after onset of genital ulcer symptoms leads to a high potential for transmission to sexual partners. This, combined with the fact that many patients with GUs delayed seeking care, shows a need for awareness campaigns about GUs and the importance of abstinence or use of condoms when experiencing such symptoms.

## Background

Sexually transmitted Infections (STIs) remain a public health problem in developing countries [1]. The common causes of genital ulcers (GUs) include Herpes Simplex Virus type 2 (HSV-2), *Treponema pallidum*, which causes syphilis, and *Haemophilus ducreyi* which causes chancroid [2-7]. Genital ulcers are particularly associated with increased risk of Human Immuno-deficiency Virus (HIV) acquisition and transmission [8], but may also lead to complications of their own, such as chronic infection, still-births and congenital infection with syphilis [9]. A meta-analysis showed a 3–4fold increase in susceptibility to HIV infection among women and men with Genital Ulcer Disease (GUD) [10]. In a Ugandan cohort of HIV discordant couples, the probability of HIV transmission per coital act increased 2fold in the presence of GUD, whereas there was no increased risk of transmission for non-ulcer syndromes [11]. Biologically the disrupted genital mucosa or skin provides a port of entry for transmission for the virus, increasing the susceptibility or infectiousness of HIV [8,12,13].

Transmission dynamics in a population are dependent upon the rate of new infections of STIs (the reproductive rate), which is a function of the probability of transmission of a pathogen, the rate of partner change, and the duration of infectiousness [14]. The probability of transmission depends on individual factors such as risky sexual behaviour, virulence and efficiency of the organism and the duration of infection [15]. Based on mathematical models, the transmission probabilities of chlamydial infection, syphilis, chancroid and HSV-2 per unprotected sexual act (in a long-term relationship involving uncircumcised men) are estimated at 0.16, 0.18, 0.2 and 0.0009 for male-to-female transmission, and 0.12, 0.15, 0.2 and 0.00015, for female-to-male transmission, respectively, if neither partner is immuno-compromised [16]. The rate at which partners are changed and the probability of transmission can be targeted partly through preventive campaigns and partner notification. Since many STIs are easily curable, onward transmission can be prevented by prompt treatment of the infected (source) patient, tracing of partner(s) within a short period, and treatment of infected or exposed partners.

Not all individuals with an STI are aware of their infection, and an asymptomatic presentation is one of the reasons for the lack of awareness [17]. Amongst those aware of the STI, some seek treatment whilst others do not [9]. A cross-sectional study of residents in Lusaka in the 1990s showed diverse preferences when seeking care for STIs. These included traditional healers, chemists, and public and private health facilities [18]. Stigma, lack of privacy and confidentiality, the availability of other care options, and perceived poor quality of care, were among the factors impeding care-seeking for STIs from public facilities [18,19]. Early diagnosis and treatment of STIs is important for comprehensive management [20] and prevents further spread. Delayed care prolongs the duration of infection [14,21] and thus increases the likelihood of its secondary transmission and the occurrence of complications, such as still births or congenital infections (associated with a high mortality) [22].

We found no recent studies in Zambia examining sexual behaviour and care-seeking among patients with GUs. This paper examines the risk factors of self-reported GUs and care-seeking in the general population, and assesses GU-patients regarding their past care-seeking, recent sexual behaviour and partner awareness of the disease.

## Methods

Two data sources were used; data from a GU aetiological study (referred to as the “GUD study”), and data from the 2007 Zambia Demographic and Health Survey (DHS). The primary objective of the GUD study was to ascertain the aetiological pattern of GUs among patients in primary health care facilities in Lusaka. Ten health centres in Lusaka district with the highest STI incidence during the year preceding the study were conveniently selected. Incidence was measured as diagnosed STI cases per 1000 inhabitants in each catchment area in 2009 according to the District Health Management Information System. Consenting patients aged 16 and above presenting with GUs were recruited consecutively between April and May 2010. A structured questionnaire which included questions on demographic data, sexual behaviour and partner’s awareness of the ulcer was administered by the research assistants. A physical examination of the genitalia was done and in addition, women had a speculum examination. Swabs obtained from the GUs were sent for Polymerase Chain Reaction (PCR) testing to detect the aetiological agent*.* Specimen handling and testing are described elsewhere (paper in press). In addition, consenting patients were given an Oral Mucosal Transudate (OMT) HIV test, using the Oraquick^®^ rapid HIV 1/2 antibody test.

The DHS data was based on 2-stage random cluster sampling. The sampling frame from the census of population and housing of Zambia for the year 2000 was used, which consists of >16,000 Standard Enumeration Areas (SEAs). A SEA is a small geographic unit consisting of 130 households on average. An urban–rural stratification was done for each of the 9 provinces of Zambia, making a total of 18 strata. In the first stage, 320 SEAs were sampled from these strata with proportional probability. In the second stage, 25 households were randomly picked from each selected SEA using equal probability systematic sampling. Men aged 15–59 and women aged 15–49 years of permanent residence or visiting the night before were eligible for interview. The overall response rate was 98%. The interviews included various questions, however of interest to our study were the questions on demographic, socioeconomic position factors, episodes of genital ulcer in the previous year, and healthcare seeking related to these episodes. The socioeconomic position indicators included were education and wealth index. Wealth index was recorded from 5 to 3 categories i.e. poorest/poorer, middle and richer/richest. All respondents were asked to consent to HIV testing and one third to syphilis testing. Serial testing using 2 ELISA tests, Vironostika^®^ HIV Uni-Form II Plus O, Biomerieux and Anti-HIV 1/2 Plus, by Enzygnost^®^ Dade Behring, was done and Western Blot 2.2, Abbott Labs, as a tie breaker. For syphilis testing, Rapid Plasma Reagin was used for screening, and Treponema Pallidum Haemagluttination Assay as a confirmatory test. Further details are described elsewhere [23].

### Data analysis

All analyses were conducted using the statistical software for social sciences, PASW for Windows version 18.0. The design effect (stratified cluster sampling) and weighting for differential selection and response probabilities were taken into account by using complex sample analyses for the DHS data. Associations were assessed for predictors of genital ulcers, care-seeking in general and late care-seeking using logistic regression models. Late care-seeking among patients in the GUD study was defined as having an ulcer for >2 weeks before care was sought. Since there was interaction between the effect of sex and the sexual behaviour variables on the risk of reported genital ulcer in the preceding year, stratified risk factor analyses for GU by sex were done. Factors that were associated (*p*-value <0.2) with reported genital ulcers in either men or women were included in a multivariate regression model. The proximate determinants conceptual framework was used to guide the analysis of predictors of genital ulcers and a hierarchical multiple regression model was built. In model I, we included socio-demographic factors, i.e. age, marital status, socioeconomic position indicated by educational attainment and wealth index which are considered underlying determinants of STIs. In model II, we added the proximate determinants (“recent sexual activity”, “type of relationship with the previous sexual partner”, “use of condom at the previous sexual intercourse”, “age at first sex” and “total number of life-time sex partners”). Only factors that were significant (*p* < 0.1) in model II were included in the final model III.

In the GUD study, there was no direct question on sex after onset of symptoms. A new variable was formed by combining information on the duration of the ulcer with the time since the most recent sexual intercourse. Both these questions had the following responses “1–6 days”, “1–2 weeks”, “>2 weeks”, “>1 month”, or “>2 months”. A person was considered to have had sex after onset of symptoms if he/she reported sex within the same period that the ulcer occurred. The question on patients` awareness of the cause of the genital ulcer was open-ended. We re-coded it into a dichotomous variable and grouped all responses that indicated attribution to having had unprotected sex with a partner who was supposedly unwell as being aware of the cause of genital ulceration. A common response for those categorised as unaware of the cause of the ulcer was witchcraft.

### Ethical considerations

The protocol for the GUD study was cleared by the Biomedical Research and Ethics Committee of the University of Zambia and the regional ethical committee of Western Norway. The Ministry of Health in Zambia gave authority to carry out the study in the designated health facilities. The patients were seen by a clinician and treated according to the national treatment guidelines for STIs based on syndromic management, before being recruited into the study. All the patients were counselled and referred for HIV testing based on an opt-out principle. Written informed consent to participate and separate written consent for HIV testing were obtained from all patients prior to participation.

Approval for the serial DHS was given in 1992 by the government through Ministry of Finance. The anonymous linked protocol developed by Measure DHS was used for HIV specimen collection and analysis, which allows linking of the results to socio-demographic data if information that potentially identifies an individual is destroyed. Information on testing, storage and further testing of the DBS was provided to all participants. Results of the HIV test were not provided, but print material was availed to all on HIV/AIDS and the available fixed points for HIV testing [23]. A single injection of the standard treatment in Zambia, Benzathine penicillin, was given to respondents that tested positive for syphilis. Alternative treatment for pregnant women and patients allergic to penicillin was provided, and referral to the nearest health facility for those who did not wish to be treated at home [23].

## Results

### DHS findings

The number of respondents who reported symptoms of GU in the general population was 409 out of 11,282 who responded to the question. The data showed that 4.1% and 3.2% sexually active men and women respectively reported having experienced GU symptoms in the previous 12 months. Multivariate analysis (model III), showed that age 25–29 years, being widowed/ separated/divorced and total number of life-time sex partners were significant predictors of genital ulcers symptoms among sexually active men in general population (Table 1). Among the sexually active women, only age 25–29 years, and increasing total number of life-time sex partners were significant predictors of GU as seen in model III (Table 2).

**Table 1:** Predictors of genital ulcer symptoms among men in the general population

	**Univariate**				**Multivariate**		
				***p*-value**	**Model I**	**Model II**	**Model III**
**Age in years**	n	%	OR (CI)	**<0.001**	OR (CI)	OR (CI)	OR (CI)
15–19	673	2.5	0.9 (0.43, 1.72)		1.1 (0.44–2.58)	1.6 (0.59, 4.33)	1.5 (0.61–3.56)
20–24	923	4.2	1.5 (0.82–2.57)		1.7 (0.85–3.23)	1.5 (0.74–3.20)	1.9 (0.99–3.67)
25–29	969	6.6	2.3 (**1.40–3.93**)		2.5 **(1.43–4.32)**	2.5 **(1.39–4.51)**	2.7 **(1.53–4.69)**
30–39	1662	3.8	1.3 (0.80–2.19)		1.3 (0.80–2.21)	1.3 (0.75–2.19)	1.4 (0.83–2.99)
40–49	862	2.9	Ref		Ref	Ref	Ref
**Residence**				0.330			
Urban	2149	4.6	1.3 (0.86–1.82)				
Rural	2940	3.7	Ref				
**Marital**				**0.038**			
Never married	1636	3.7	Ref		Ref	Ref	Ref
Married / Cohabiting	3777	3.9	1.1 (0.72–1.59)		1.2 (0.65–2.14)	0.8 (0.27–2.31)	0.8 (0.49–1.49)
Widowed /Separated / Divorced	276	8.5	2.4 (**1.47–4.07**)		**2.6 (1.35–5.02)**	2.2 **(1.04–4.84)**	2.2 **(1.13–4.20)**
**Wealth index**				**0.176**			
Poorest / Poorer	1705	3.6	Ref		Ref	Ref	
Middle	1048	3.4	0.9 (0.60–1.50)		0.9 (0.58–1.45)	0.9 (0.56–1.45)	
Richer / Richest	2338	4.7	1.3 (0.90**–**1.94)		**1.3 (0.85–1.89)**	1.2 (0.78–1.79)	
**Education**				0.304			
No education	246	4.6	Ref				
Primary	2331	4.4	0.9 (0.49–1.82)				
Secondary/ Tertiary	2512	3.8	0.8 (0.41–1.60)				
**Recent sexual activity**							
Active last 4 weeks	3135	4.7	1.6 **(1.07–2.29)**	**0.010**		1.5 (0.944–2.36)	1.7 (1.07–2.61)
Not active last 4 weeks	1948	3.1	Ref			Ref	Ref
**Used condom last sex**				**0.043**			
No	3375	4.1	Ref			Ref	
Yes	1066	5.5	1.4 (0.92–2.01)			0.9 (0.60–1.36)	
**Relationship with last partner**				**0.015**			
Spouse/Live-in partner	3107	4.0	Ref			Ref	
Casual acquaintance /Girl friend	1301	5.5	1.4 (**1.01–1.95**)			0.9 (0.38–2.26)	
**Age at first sex (in years)**				**0.065**			
<16	1923	4.9	1.3 (0.95–1.77)			1.0 (0.63–1.55)	
16–19	2094	3.8	1.1 (0.81–1.52)			0.8 (0.48–1.27)	
>19	1072	3.3	Ref			Ref	
**Life-time sex partners**				**<0.001**			
1	771	1.0	Ref			Ref	Ref
2–4	2223	3.2	3.2 **(1.34–7.73)**			3.5 **(1.22–9.87)**	3.0 **(1.27–7.08)**
5–10	1452	6.2	6.3 **(2.68–14.94)**			6.4 **(2.32–17.94)**	5.7 **(2.39–13.33)**
11 and above	636	6.8	7.0 **(2.85–17.52**)			7.2 **(2.40–21.40)**	6.1 **(2.42–15.28)**
**Circumcised (men only)**				0.966			
No	4212	4.0	0.83 (0.56–1.22)				
Yes	876	4.8	Ref				

Model 1 Underlying variables with *p* < 0.2 in the univariate analyses.

Model 2 Underlying + proximate determinants with *p* < 0.2 in the univariate analyses.

Model 3 variables with *p* < 0.1 in model II.

**Table 2:** Predictors of genital ulcer symptoms among women in the general population

	**Univariate**				**Multivariate**		
				***p*-value**	**Model I**	**Model II**	**Model III**
**Age in years**	n	%	OR (CI)	**<0.001**	OR (CI)	OR (CI)	OR (CI)64
15–19	790	2.3	1.0 (0.52–2.02)		1.3 (0.64–2.64)	1.6 (0.81–3.31)	1.3 (0.–2.55)
20–24	1307	2.8	1.3 (0.74–2.24)		1.4 **(**0.81–2.55)	1.6 (0.87–2.79)	1.4 (0.81–2.50)
25–29	1348	4.5	2.1 (**1.26–3.46**)		2.2 **(1.32–3.74)**	2.3 **(1.33–3.80)**	2.2 **(1.30–3.61)**
30–39	1763	3.5	1.6 (0.94–2.84)		1.7 (0.98–2.94)	1.7 (0.98–2.95)	1.67 (0.96–2.89)
40–49	985	2.2	Ref		Ref	Ref	Ref
**Residence**				0.248			
Urban	2605	3.6	1.2 (0.87–1.73)				
Rural	3588	3.0	Ref				
**Marital**				**0.027**			
Never married	1002	2.4	Ref		Ref	Ref	
Married / Cohabiting	4302	3.2	1.4 (0.85–2.20)		1.4 (0.82–2.27)	1.5 (0.93–2.49)	
Widowed /Separated / Divorced	889	4.1	1.8 (**1.01–3.15**)		1.8 (0.98–3.34)	1.7(0.93–3.03)	
**Wealth index**				**0.051**			
Poorest / Poorer	2185	2.7	Ref		Ref		
Middle	1274	3.0	1.1 (0.74–1.73)		1.1(0.74–1.73)	1.1 (0.74–1.72)	
Richer / Richest	2734	3.8	1.4 (0.96–2.06)		1.4 (0.98–2.10)	1.4 (0.98–2.09)	
**Education**				0.303			
No education	714	2.4	Ref				
Primary	3426	3.3	1.4 (0.73–2.53)				
Secondary/ Tertiary	2053	3.4	1.4 (0.76–2.72)				
**Recent sexual activity**							
Active last 4 weeks	3767	3.4	1.0 (0.73–1.49)	0.893			
Not active last 4 weeks	1609	3.3	Ref				
**Used condom last sex**				0.693			
No	4615	3.4	Ref				
Yes	691	3.7	1.1 (0.67–1.82)				
**Relationship with last partner**				0.322			
Spouse/Live-in partner	4339	3.3	Ref				
Casual acquaintance /Girl friend	940	4.1	1.3 (0.85–1.86)				
**Age at first sex (in years)**				0.613			
<16	2326	3.4	1.2 (0.72–1.84)				
16–19	2783	3.2	1.1 (0.68–1.79)				
>19	1084	2.9	Ref				
**Life-time sex partners**				**<0.001**			
1	2740	2.0	Ref				
2–4	3115	4.0	2.0 (**1.42–2.90**)			2.0 **(1.37–2.79)**	2.0 **(1.37–2.82)**
5–10	298	7.4	3.9 (**2.21–6.91**)			3.9 **(2.17–7.01)**	3.9 **(2.18–6.93)**
11 and above	33	7.9	4.2 (**0.90–19.29**)			4.6 (0.93–22.49)	4.5 (0.95–21.70)

Model 1 Underlying variables with *p* < 0.2 in the univariate analyses.

Model 2 Underlying + proximate determinants with *p* < 0.2 in the univariate analyses.

Model 3 variables with *p* < 0.1 in model II.

Most respondents (78%) had sought care for their ulcers. Of these, 60% had sought care at a public health facility, 10% at a private and 30% at another kind of facility. There was no association between residence, socio-economic position or sex and seeking care for GU symptoms in the general population. (Additional file 1: Table S1).

### Findings from the GUD study

The mean age of the enrolled patients with genital ulceration in Lusaka was 28 years. The male patients were older and had higher level of education compared to the females. About 14% of the patients had ulcers that had lasted >2 weeks before seeking care. Nearly 60% of the patients reported that their partners knew about their genital ulcers. Male patients were more likely to be aware of the cause of genital ulcers whereas the females were more likely to be aware of their HIV status (Table 3). The awareness of HIV status was particularly high among HIV positive women (77%) compared to HIV positive men (60%). Half the patients with GUD that consented to saliva-based HIV testing were HIV infected. Most of those who had previously sought care for the present ulcer had attended a government clinic. Men were more likely to have sought previous care for the current ulcer at a private clinic than women (Table 3).

**Table 3:** Characteristics of patients with GUD attending government clinics in Lusaka, Zambia

	**Overall no. of patients (%)**	**Males n (%)**	**Females n (%)**	***p*-value**
**Age** Mean, in years (SD)	28.18 (7.4)	29.35 (7.5)	27.01 (7.2)	**0.013**
**Education: (n = 201)**				**0.002**
Primary / no education	84 (41.8 )	31 (31)	53 (52.5)	
Secondary / tertiary	117 (58.2 )	69 (69)	48 (47.5)	
**Duration of ulcer**				0.638
1–6 days	61 (30.8)	28 (28)	33 (30.8)	
1–2 weeks	55 (27.8)	31 (31)	24 (27.8)	
more than 2 weeks	19 (9.6)	11 (11)	8 (9.6)	
more than 1 month	16 (8.1)	9 (9)	7 (8.1)	
more than 2 months	47 (23.7)	21 (21)	26 (23.7)	
**When last did you have an ulcer? (n = 96)**				0.842
Last 3 months	41 (42.7)	21 (40.4)	20 (45.5)	
Last 6 months	19 (19.8)	12 (23.1)	7 (15.9)	
Last 1 year	10 (10.4)	5 (9.6)	5 (11.4)	
More than 1 year	26 (27.1)	14 (26.9)	12 (27.3)	
**Partner aware of your genital ulcer (n = 195)**				0.240
Yes	113 (57.9)	51 (53.7)	62 (62.0)	
No	82 (42.1)	44 (46.3)	38 (38.0)	
**Knowledge on cause of genital ulcers (n = 184)**				**0.008**
Have knowledge	90 (48.9)	55 (58.5)	35 (38.9)	
Do not have knowledge	94 (51.1)	39 (41.5)	55 (61.1)	
**Aware of their HIV status (n = 125)**				0.076
Yes	73 (58.4)	29 (50.0)	44 (65.7)	
No	52 (41.6)	29 (50.0)	23 (34.3)	
**HIV Status (n = 128)**				0.730
Positive	63 (49.2)	32 (47.8)	31 (50.8)	
Negative	65 (50.8)	35 (52.2)	30 (49.2)	
**Sought treatment previously for present ulcer (n = 177)**				0.727
**Yes**	101 (57.1)	52 (57.8)	49 (56.3)	
**No**	76 (42.9)	38 (42.2)	38 (43.7)	
**Where (n = 101)**				0.07
Government hospital/health centre	70 (69.3)	31 (59.6)	39 (79.6)	
Private hospital/health centre	16 (15.8)	12 (23.1)	4 (8.2)	
Traditional healer	15 (14.9)	9 (17.3)	6 (12.2)	
**Sex since onset of symptoms**				0.950
No	82 (42.9)	41 (43.2)	41 (42.7)	
Yes	109 (57.1)	54 (56.8)	55 (57.3)	
**Condom-use since ulcer developed**				0.071
Always	21(15.0)	12 (17.0)	9 (13.0)	
Sometimes	41 (28.0)	16 (22.6)	25 (33.3)	
Never	123 (57.0)	65 (60.4)	58 (53.7)	

*GUD* Genital Ulcer Disease.

*SD* Standard deviation.

We also found that patients with bilateral nodes and recent experience of ulcers were less likely to have postponed care-seeking than those with unilateral nodes (7 vs. 21%) and first occurrence of an ulcer (9 vs. 16%), respectively. The differences were however non-significant. The data showed no association between education or severe symptoms of genital ulcers such as bleeding, pain, or genital swelling and late care-seeking (results not shown).

About 60% of the GUD patients in Lusaka reported that their partners knew about their genital ulcers. The data indicated that patients with higher education were more likely to have partners who were aware of the ulcer compared to their less educated counterparts (*p* = 0.06, i.e. borderline significant), and those who had been sexually active after the onset of symptoms were more likely to have partners who were aware of the ulcer (Table 4). Fifty-seven percent reported sex after onset of symptoms, and consistent condom use was reported by only 15% of them. Among those whose partners were aware of the ulcer, 16% reported consistent condom use versus 12% of those whose partners were unaware (*p* = 0.837).

**Table 4:** Probability of GUD patients reporting that partners are aware of their STI

	**n**	**%**	***P*-value**
**Age**			
15–19	25	48.0	0.689
20–24	43	62.8	
25–29	49	61.2	
30–39	40	57.5	
40–49	37	51.4	
**Sex**			
Male	95	53.7	0.330
Female	99	60.6	
**Education**			
0–7 years	81	49.4	0.062
>7 years	113	62.8	
**Sex after onset of symptoms**			**0.001**
No	80	43.8	
Yes	108	68.5	
**Knowledge on causes of genital ulcers**			
Knowledgeable	90	52.2	0.206
Not knowledgeable	91	61.5	

*GUD* Genital Ulcer Disease.

## Discussion

The data from the Zambia DHS 2007 indicated a low annual prevalence of genital ulcers in the general population. We found that the majority of the respondents sought care in public health facilities in contrast to earlier studies from the late 1990s, that showed a preference for private over public facilities [18,19]. There were no differences by residence or socioeconomic position indicators in care-seeking among respondents in the general population. The data from the GUD study in Lusaka indicated that most patients with genital ulcers were sexually active after the onset of symptoms and that consistent condom use was very low. This indicates a great potential for further transmission of the disease, while partner awareness had a disappointingly limited impact on condom use.

The age category with the highest estimate of GU symptoms in both men and women in the general population was 25–29 years. This could reflect higher sexual activity in this age group although the risk remained significantly higher even after adjusting for some sexual behaviour characteristics. Some factors such as concurrency, frequency of sex or consistent use of condoms, however could not be adjusted for as they were not available, and thus could explain the persistent association.

Widowhood, separation or being divorced was also associated with GU symptoms in men, although this was not the case in women. It is possible that the widowed and separated/divorced engage in more risky sexual behaviour, putting them at risk of GUD, as seen in a Zimbabwean study where widows and widowers had more partners, a higher partner change rate and were likely to engage in transactional sex, compared to the non-widowed [24]. We also found that HIV prevalence was significantly higher among this group, which is in line with other studies [24-26]. This could be due to higher risky sexual behaviour among them or could arise from the fact that the most common cause of adult mortality in Sub-Saharan Africa is HIV. If the deceased spouse died of HIV, the widowed respondent would obviously be at increased risk of being infected too [24,27].

The prevalence of GUD was the same irrespective of education status. This does not correspond with the findings from 5 rounds of syphilis surveillance among pregnant women in Zambia between 1994–2008 which showed consistently lower risk of syphilis in higher educated women [28]. It is likely that GUD is substantially under-reported in this population-based survey, and therefore this lack of association could be explained by differential reporting bias, i.e. that under-reporting is negatively associated with level of education due to a higher level of awareness among the higher educated. Under-reporting could also be due to both recall and social desirability biases, and may result in no apparent associations, especially for small samples and rare conditions, as in this case.

In this study, the majority of the respondents from the general population sought care from a public health facility, but 40% sought care from private or “other” unspecified facilities. “Other” facilities comprised of a shop or pharmacy and the non-biomedical sector. Alternative sources for STI care reported previously in Zambia and other African countries include chemists/pharmacies and traditional healers [18,19,29,30]. Reasons cited in literature, why patients seek care outside the public sector, include long waiting time at the health facilities, lack of privacy, lack of drugs and insistence on bringing the sexual partner(s) for treatment [18,19,31,32]. Although the quality of services varies in both private and public health facilities, there are reports that suggest that the latter provide better STI services [17,33-35]. Public facilities may be better placed in providing good services if a functional national STI programme is in existence, which involves in-service trainings, provision of treatment guidelines and ensuring constant drug supplies for syndromic management of STIs. Literature shows that many private facilities fall short of standardised care [34] as seen in a South African study in Hlabisa, which compared STI therapeutic practices of private doctors with standard guidelines from the provincial health department. The treatment of STIs by the private doctors was not in line with provincial guidelines. In that study, patients were inadequately treated and a variation was observed in the type of treatment prescribed for any single syndrome among the doctors [36]. In Zambia, periodic performance appraisals are conducted for all public facilities by supervising authorities. Public facilities also benefit from capacity building programmes organised by the national control programme, while private facilities are often not included in these quality assurance mechanisms. Considering the different players involved in care for STIs, standardised STI care in both public and private facilities should be an important strategy in reducing STI morbidity. Another key strategy is to increase awareness among the traditional healers on STIs, their consequences, and the need to refer patients to health facilities where effective treatment can be obtained. Encouragingly, only a minority of the patients from the GUD study in Lusaka sought care >2 weeks after symptom onset. It was however surprising that the DHS data showed that there were only minor differences in care seeking by residence and socioeconomic position indicators. Considering that only 50% of the rural population in Zambia live within 5 km of a health facility, compared to >90% of the urban population, one would have expected a difference in the likelihood of seeking care between urban and rural residents [37]. The data suggest that there are no substantial inequities to care seeking in this setting. This finding should be interpreted with caution since the association might be affected by the likely substantial under-reporting of symptoms.

In the GUD study, almost half the patients had partners who were aware of their STI. The association found between partner awareness and higher likelihood of engaging in sex after onset of symptoms may indicate that the ulcer had become visible during intercourse. However, it is surprising that only 16% of those who had partners who were aware of the ulcer used condoms consistently. Condom use in Africa is still generally low [34,38] and having sex while having symptoms of an STI also seem to be common as seen in other studies too [30,39,40]. These findings indicate that partner notification may only stop transmission if the partner is successfully treated, since awareness of an ulcer may not result in preventive precautions. Partner notification and treatment, and patient counselling and advice to inform their partners to seek care are all included in the Zambian STI guidelines. However, there is no legal framework-backing for this, and the notification system does not function optimally [41]. Although notification and referral to facilities for treatment may deter some patients from seeking care at government facilities [19], it remains important for reducing secondary spread and re-infections. However, patients` unwillingness to disclose all partners, can impede such notification programmes, and tracing partners without the assistance of the source patient is time-consuming [41]. Of concern was also the high proportion of GUD patients who did not know their HIV status, considering the increased risk of such patients to acquire or transmit HIV to their partners. This is compounded by low condom use and tendency to continue having unprotected sex while symptomatic. Control programmes must attempt to ensure that most GUD patients are tested for HIV and risk reduction messages are re-enforced.

In addition to the previously discussed weakness of reporting bias in self-reporting of STIs in the population-based surveys, some other important limitations of our study are worth considering. The sample for the GUD study was small and designed for another study to ascertain microbiological etiologies of genital ulcers in Lusaka using molecular techniques. The small sample therefore limited the ability to establish significant differences between the sub-populations. The study was facility-based, thus representing GUD patients who seek care in primary healthcare facilities and do not necessarily represent all STI patients and those who sought care elsewhere. On the other hand, a strength worth noting is the representativeness of the DHS surveys at national, provincial, and rural/urban levels of the country and the overall high response rate of the 2007 survey. Non-response in the DHS was mainly due to absence, while refusal to participate was low [23]. However, the low number of people reporting GU symptoms meant that only a small sample was available when examining predictors of care-seeking, resulting in few significant associations, although the percentages indicated important differences between subgroups.

## Conclusions

In conclusion, public awareness needs to be raised on the importance of early treatment seeking at public facilities, long-term consequences of STIs and sexual risk reduction as strategies for reducing STIs. Intensification of health campaigns on abstinence and promoting condom-use when there is ulceration are key to stopping further transmission of infections. Partnering with providers in the private sector ought to be considered to ensure referral where appropriate, and standardised patient care.

## Competing interests

Authors declare no competing interests.

## Authors’ contributions

MM also participated in the fieldwork of the GUD study. All authors participated in the conception, design, and analysis, preparation of the manuscript and reading and approval of the final version.

## Pre-publication history

The pre-publication history for this paper can be accessed here:

http://www.biomedcentral.com/1471-2458/12/407/prepub

## Supplementary Material

Additional file 1 Predictors for care seeking among respondents with self reported GUD in the general population in Zambia. Click here for file
