# Supplementary material for: Risk factors, healthcare-seeking and sexual behaviour among patients with genital ulcers in Zambia
Source: BMC Public Health. 2012 Jun 6;12:407. doi: 10.1186/1471-2458-12-407 (PMC3490779; doi:10.1186/1471-2458-12-407)
Supplement: Additional file 1 — Predictors for care seeking among respondents with self reported GUD in the general population in Zambia. [file 1471-2458-12-407-S1.doc]

**Table 1 (supplementary)**

**Predictors for care seeking among respondents with self reported GUD in the general population in Zambia**

| **Age (years) (398)** | n | % | OR | CI | p-value |
| --- | --- | --- | --- | --- | --- |
| 16-20 | 33 | 84.9 | 1.5 | 0.44 – 5.08 | 0.103 |
| 20-24 | 76 | 77.9 | 0.9 | 0.34 – 2.60 |  |
| 25-29 | 116 | 69.4 | 0.6 | 0.24 – 1.56 |  |
| 30-39 | 125 | 83.2 | 1.3 | 0.50 – 3.49 |  |
| 40-49 | 48 | 78.9 | Ref |  |  |
| **Sex (398)** |  |  |  |  | 0.707 |
| Male | 196 | 78.6 | 1.1 | 0.64 – 1.94 |  |
| Female | 202 | 76.8 | Ref |  |  |
| **Residence (398)** |  |  |  |  | 0.932 |
| Urban | 189 | 77.4 | 0.9 | 0.49 – 1.92 |  |
| Rural | 209 | 77.9 | Ref |  |  |
| **Education (398)** |  |  |  |  | 0.601 |
| No education | 27 | 73.4 | Ref |  |  |
| Primary | 211 | 76.1 | 1.2 | 0.40 – 3.28 |  |
| Secondary | 160 | 80.6 | 1.5 | 0.50 – 4.50 |  |
| **Marital (398)** |  |  |  |  | 0.143 |
| Never married | 85 | 80.5 | Ref |  |  |
| Married / Cohabiting | 254 | 79.3 | 0.9 | 0.44 – 1.96 |  |
| Widowed /Separated / Divorced | 59 | 67.3 | 0,5 | 0.22 – 1.16 |  |
| **Wealth (398)** |  |  |  |  | 0.953 |
| Poorest / Poorer | 115 | 76.7 | Ref |  |  |
| Middle | 75 | 77.3 | 1.0 | 0.48 – 2.24 |  |
| Richer / Richest | 208 | 78.4 | 1.1 | 0.54 – 2.25 |  |
| **Insurance cover (398)** |  |  |  |  | 0.151 |
| No | 349 | 76.4 | 0.5 | 0.17 – 1.32 |  |
| Yes | 49 | 87.2 | Ref |  |  |
| **Working (392)** |  |  |  |  | 0.601 |
| No | 121 | 79.6 | 1.2 | 0.67 – 2.02 |  |
| Yes | 272 | 77.1 | Ref | 0.50 – 1.50 |  |
| **Recent sexual activity (382)** |  |  |  |  | 0.722 |
| Active last 4 weeks | 265 | 77.6 | 1.1 | 0.56 – 2.27 |  |
| Not active last 4 weeks | 117 | 75.3 | Ref |  |  |
| **Used condom last sex (364)** |  |  |  |  | 0.351 |
| No | 282 | 76.5 | 0.7 | 0.33 – 1.48 |  |
| Yes | 82 | 82.3 | Ref | 0.68 – 3.01 |  |
| **Age first sex (398)** |  |  |  |  | 0.531 |
| 15 years or less | 163 | 78.3 | 1.5 | 0.69 – 3.12 |  |
| 16-19 years | 170 | 79.8 | 1.6 | 0.68 – 3.75 |  |
| ≥20 years | 65 | 71.2 | Ref |  |  |
| **Life-time sex partners (398)** |  |  |  |  | 0.358 |
| 1 | 64 | 72.3 | Ref |  |  |
| 2-4 | 191 | 75.9 | 1.2 | 0.63 -2.30 |  |
| 5-9 | 103 | 81.3 | 1.7 | 0.80 – 3.47 |  |
| ≥10 | 40 | 85.4 | 2.2 | 0.70 – 7.09 |  |
| **Syphilis test result (105)** |  |  |  |  | 0.371 |
| Negative | 95 | 69.5 | Ref |  |  |
| Positive | 10 | 83.0 | 2.2 | 0.39 – 11.69 |  |
| **HIV test result (345)** |  |  |  |  | 0.231 |
| Negative | 221 | 75.1 | Ref |  |  |
| Positive | 124 | 79.5 | 1.3 | 0.78 – 2.12 |  |
|  |  |  |  |  |  |

GUD-Gental Ulcer Disease
